# Supplementary figures and images for: Distinct alpha networks modulate different aspects of perceptual decision-making
Source: PLoS Biol. 2025 Oct 22;23(10):e3003461. doi: 10.1371/journal.pbio.3003461 (PMC12561919; doi:10.1371/journal.pbio.3003461)

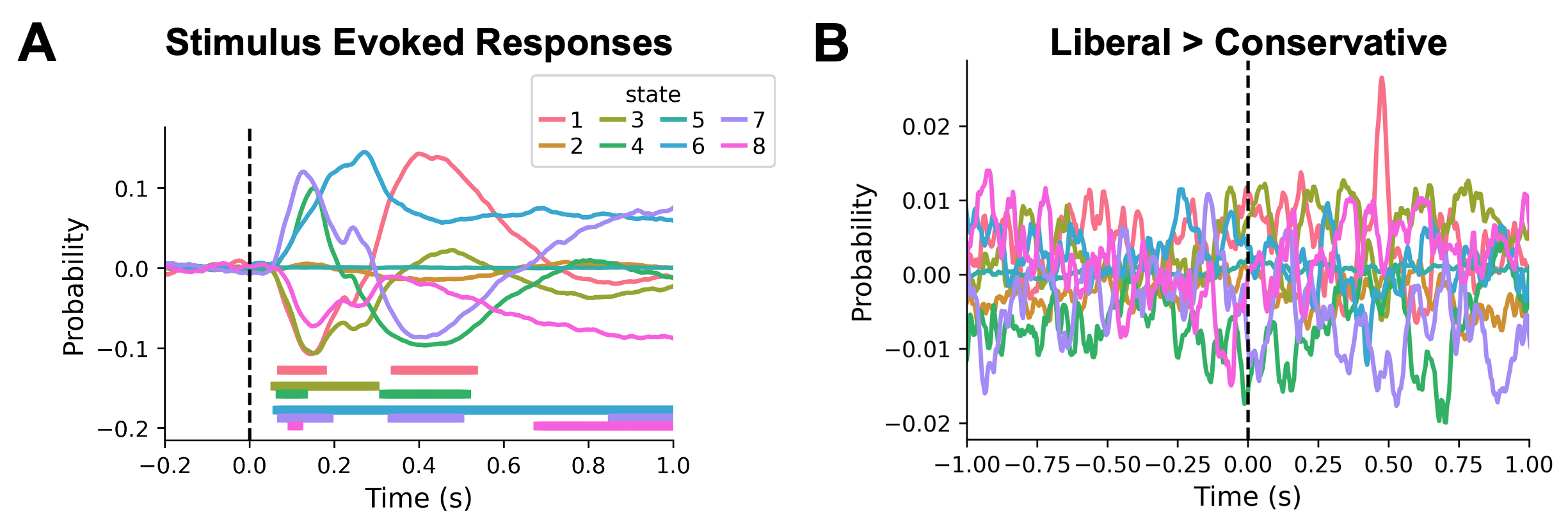

Supplement: S1 Fig — (A) State time courses epoched around the presentation of stimulus (onset denoted by the dashed black line). Horizontal bars indicate time points that contributed to the significant clusters. (B) Difference state time courses between the liberal and conservative conditions. Data underlying this Figure can be found in S1 Data. (TIFF) [file pbio.3003461.s001.tiff]

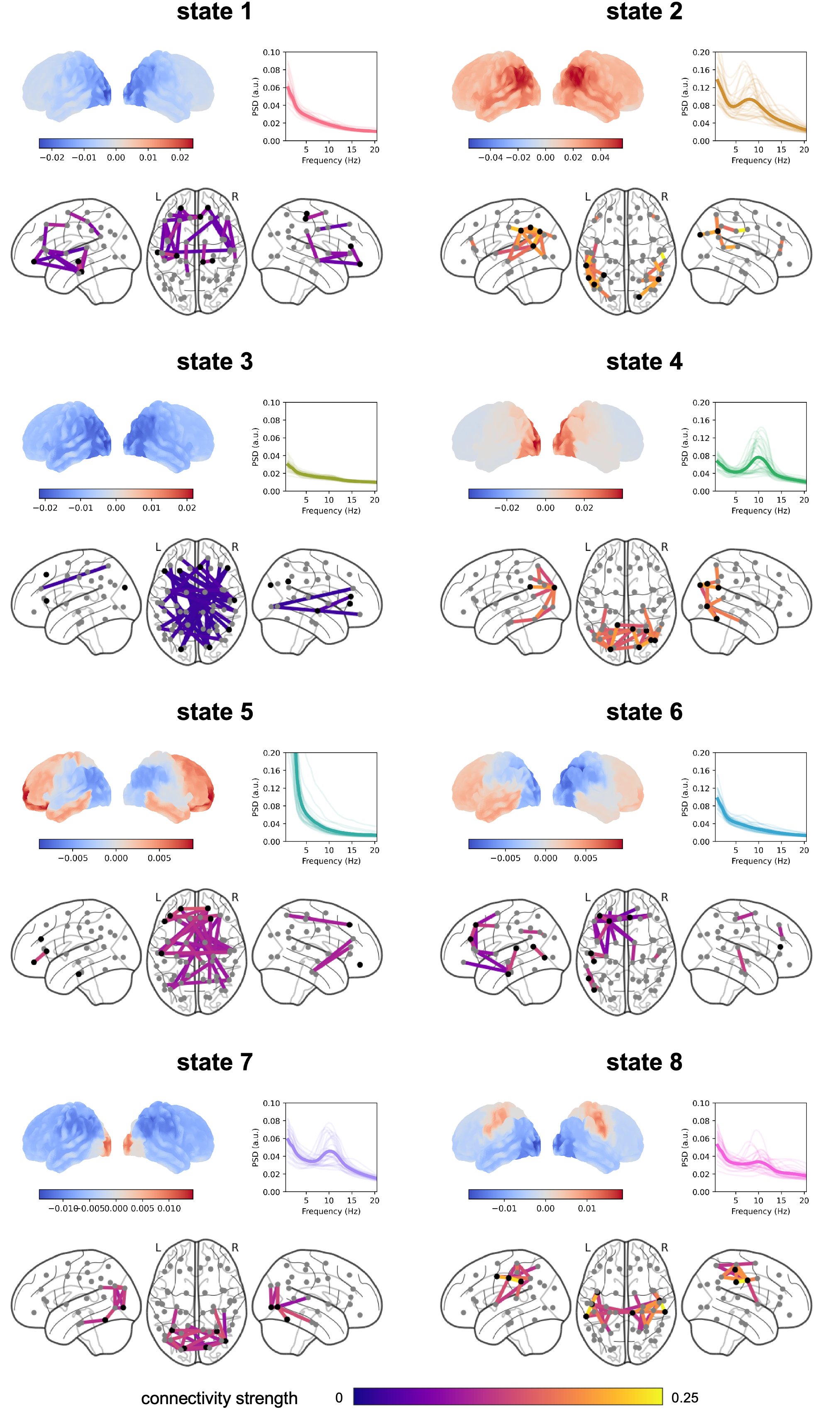

Supplement: S2 Fig — Sub-panels are organized similarly to Fig 2; the PSD graph (top right) shows the state-specific PSD (and the corresponding standard error, shaded area) estimated from the critical nodes (highlighted in black in the coherence network). Data underlying this Figure can be found in https://doi.org/10.5281/zenodo.17217503. (TIFF) [file pbio.3003461.s002.tiff]

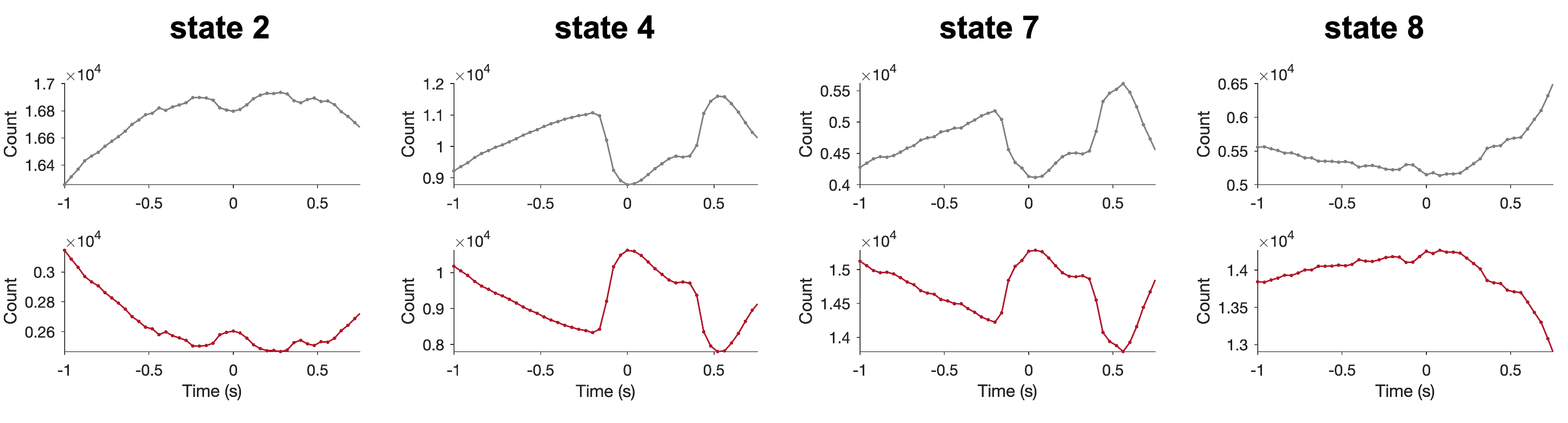

Supplement: S3 Fig — Upper row: A = 0; lower row: A = 1. Data underlying this Figure can be found in S3 Data. (TIFF) [file pbio.3003461.s003.tiff]
