# Supplementary material for: Distinct alpha networks modulate different aspects of perceptual decision-making
Source: PLoS Biol. 2025 Oct 22;23(10):e3003461. doi: 10.1371/journal.pbio.3003461 (PMC12561919; doi:10.1371/journal.pbio.3003461)
Supplement: Attachment — Submitted filename: response_to_reviewer_v3.docx [file pbio.3003461.s009.docx]

We sincerely thank the editor and reviewers for their time and effort, and we appreciate the opportunity to revise and improve our manuscript. We hereby provide the point-by-point response (in blue) to each comment, and corresponding revisions (see the ‘track-changes’ version) in the updated manuscript.

*Reviewer #1:*

The presented study investigates whether the presence of specific states exhibiting alpha-range activity impacts perceptual decision making. The methods set is advanced but straightforward and well described. The results indicate that different subprocesses residing in the alpha-range are differentially influencing upcoming perceptions. In that sense the study presents novel and intriguing results.
I have minor comments which need to be addressed to improve the presentation and reproducibility of the findings.

We thank the reviewer for their positive evaluation of our work.

The more important ones regard the specifics of the HMM implementation:
Methods, HMM, end of 1. paragraph p.16: Was the variability of the free energy taken into account across the 10 replications of the estimations? Simply taking the lowest value would not necessarily make it more robust.
How were state number and embedding number chosen? Which criteria were employed? Was any estimation performed on how a choice of different values would affect the results?

These are all great questions, and we appreciate the opportunity to provide further details on our approach. The variability of the free energy across 10 replications were not taken into account in the current case. This is because the goal of these 10 runs is to find the “best” model, reflecting the most parsimonious fit to the MEG data. To this aim, we used free energy as the primary metric for model selection. The variability of free energy across runs mainly reflects the across-run consistency of the optimization approach, but does not necessarily provide insight about the data or the true model generating the data.

That said, we fully share the reviewer’s concern regarding the robustness of the HMM results. We have taken the following approach to ensure that the HMM results are stable and robust: First and foremost, we have ensured that the input data are sufficient in size. Here, the input data consisted of >30 hours of MEG recordings, which was substantially larger in size compared to existing work using the same approach to establish networks. For example, a total of <19 hours of MEG recording was used in Gohil et al., 2024 and Quinn et al., 2018, and a total of <5 hours of MEG recording was used in Vidaurre et al., 2018. Moreover, we have repeated the inference 10 times and chosen the best run with the smallest free energy. This was considered a sufficient number of replications (Gohil et al., 2024). In fact, Gohil and colleagues (2024) performed the model-fitting procedure 3 times with 10 runs each. They showed that the inferred summary statistics for the best run from each set of 10 runs are extremely similar, indicating convergence and reproducibility of the HMM results using the current approach with 10 runs.

The state number and embedding number were chosen based on the recommendation by developers of the osl-dynamics toolbox (<https://osl-dynamics.readthedocs.io/en/latest/faq.html>). We did not go beyond 8 states like previous work (van Es et al., 2023; Vidaurre et al., 2018) for the following reasons: First, with K=8 states, we were able to observe the occipital, parietal and parietal-frontal networks as one would have expected based on previous work. Furthermore, including more states would necessarily lead to less occupancy per state, and ultimately less “state on” trials for each of the GLMM per state and per time point.

The reviewer raised an interesting point that using a different number of states may affect the results. Therefore, we refit the TDE-HMM model with K=10 and attach below the output from that with the lowest free energy across 10 replications: the spatial distribution of power estimated for [1, 45] Hz of each state (data), and the corresponding FOOOF model fit to the state-specific PSDs (in black solid lines).

Based on the PSDs, one would most likely identify state 4, 6, 8, 9, and 10 as alpha states. Yet, upon closer inspection, state 10 would likely be excluded from further analysis as its corresponding PSD falls consistently below the static PSD, making it uninterpretable. The remaining alpha states 4, 6, 8 and 9 closely resemble the spatial configurations of the alpha states identified with K=8 in our Figure 2.

Taken together, we believe the current setting of K=8 is a reasonable choice serving the purpose of identifying alpha networks and their moment-to-moment dominance status.

Other:
p. 16 last line: Was only the first mode used as in the original publication?
Spectral analysis, 2nd paragraph p.17: Was any absolute criteria regarding the relevance of the nodes was applied on top of the relative criteria? Would that affect the results?

Yes, only the first mode was used in the current work. The first mode resulted from the non-negative matrix factorization typically covers 1-25 Hz, which serves the current purpose of configuring alpha oscillatory networks.

We did not apply any additional criteria to the selected nodes on top of the relative criteria (0.25% strongest connectivity). We could potentially apply the FOOOF algorithm to the PSD of each selected parcel/node, and include only the nodes exhibiting prominent alpha oscillatory components. This could be an “absolute criterion” as the reviewer suggested. Yet we did not take this additional step because this would most likely result in a different number of nodes selected per subject per state, adding more between-subjects and between-states variation to the analysis.

Section GLMM: Why is a sliding window approach used, when responses occur at distinct time points? The answer becomes apparent in the results section. It should be made more explicit in the methods section.

We thank the reviewer for this suggestion, and added the following clarification to Figure 1’s caption:

*Grey shaded areas denote the 500-ms sliding window used for estimating* $P_{t,k}$*.*

Bottom of p.21 I don't know how this is done? Couldn't individual event-related responses be directly tested against zero in a parametric or non-parametric test?

The reviewer is correct that the individual event-related responses can be directly tested against zero (after baseline correction), which was exactly what we did. We apologize for the confusion and have rewritten this part to clarify the cluster-based permutation tests.

*To compare the liberal and conservative conditions, we permuted the condition labels of the stimulus-evoked responses 5000 times to generate a distribution of cluster-level statistics from the permuted samples. This distribution was then used for estimating the statistical significance of the real data. Similarly, to test whether stimulus-evoked responses differed significantly from baseline, we randomly shuffled the baseline-corrected data with (synthetic) time series of zeros 1000 times, and compared the cluster statistics of the real data to those from the permuted samples for calculating the statistical significance.*

Fig. 2 I don't understand the projections, particularly state 5 has many coherences from the top view, but they are not visible on the coronal or sagittal views.

Great catch! This is possible because a great number of connections in state 5 are bilateral (between the same area on the left and right hemisphere). To make it more intuitive, below we also include the coherence map of state 5 viewed from the back of the head.

Fig. 4 Actually, I don't know what measures beta_d' and beta_c are given as they do not appear in the methods section. It would be helpful if measures beta_d',A=1 and beta_d',A=0 etc would be plotted along with the significances.
Were any corrections for multiple comparisons applied? Could a measure for cross-subject error be indicated?

We apologize for the confusion. In fact, the “state status” legends at the bottom of Figure 4 denoted exactly the “A=1” and “A=0” scenarios, as the reviewer suggested. We have now further clarified the legend and also added this to the figure caption.

As for the corrections for multiple comparisons, we used a cluster-based permutation test. Specifically, time-resolved beta_d’ and beta_c values were tested, and the corresponding *F*-values were clustered along the time axis. The relevant details are provided in the *“Statistical analysis”* section.

Regarding the cross-subject error, or in other words, cross-subject variability, this was primarily accounted for by including the random factors terms in the GLMM. While the intercept and the *S* term (*S*timulus: 1 for grating and 0 for noise) were modelled as random factors, others (*P*, alpha power; *A*, state on/off status; two-way interactions between *S*, *P*, and *A*, and the three-way interaction between *S*, *P*, and *A*) were modelled as fixed factors. We could potentially show the variability of the fitted coefficients of the random factors, however these do not correspond to the between-subjects variability of beta_d’ nor beta_c. A more useful approach is to show the standard errors (SE) or the 95% confidence intervals (CI) of the fitted beta_d’ and beta_c (see figure below), yet these CIs only indicate the precision in estimating these coefficients, not exactly the cross-subject error/variability of them.

To improve readability and show variability in parameter estimation, we updated the original figure legend and added the SEs to Figure 4.

*Figure 4. Top: The critical nodes used to estimate network-level alpha activity (highlighted in different colors for visibility). Bottom: Time courses of* $\beta_{d^{'}}$ *and* $\beta_{c}$ *when the network was on (i.e., A = 1; in red) vs. off (i.e., A = 0; in grey). Horizontal bars indicate time points that contributed to significant effects (red denotes state activity, i.e., on/off, black denotes the interaction term, i.e., state on/off × alpha power). Shaded area around the lines denotes the standard error (SE) of the estimated parameters.*

Reviewer #2: Summary: This work interrogates the often-examined relation between alpha-band oscillatory dynamics and perceptual variation, using MEG to disentangle relations between perceptual sensitivity/criterion and distinct regional alpha-rhythmic patterns. The chief contribution of this paper (differentiating it from prior analyses of the same data) is the use of a data driven Hidden Markov Modeling approach to define networks with distinct spatial/spectral profiles. This work seems like a novel and useful extension of current characterizations of alpha-perception relations, adding to our understanding of regional dynamics in a way that is largely absent from existing reports, and seems likely to inspire new inquiries in this domain.
Overall, I found the approach interesting, and I do not have major concerns regarding the methodology. Most of my comments below can likely be addressed with clarification in the text, and are therefore rather minor. Thanks for the opportunity to review this work.

We appreciate the reviewer’s positive assessment and supportive comments on our work.

-
[General text]

I found the text generally well-written and clear (thanks!). I had just two related notes throughout: Given that this paper builds on prior work by utilizing a new analytic approach to address the same question in the same data (see Zhou et al., 2021), I think the writing could benefit from more elaboration regarding how these results compare with the results and conclusions of the 2021 paper. For example, rather than simply stating that "the observed sensitivity modulation by alpha activity in the visual network is consistent with our previous report" (discussion p2), it would be helpful to elaborate on what the first report found (in terms of location/timing, for example), and the similarities/differences between those observations and the results using this approach (i.e., a more explicit comparison between the two reports would provide the reader with a better understanding of the value added by adopting the current method - this is done to an extent with the criterion
effect already).

We thank the reviewer for this suggestion, and agree a more explicit comparison with our original work would be beneficial. In the Discussion, we have now added the relevant contents:

*The observed sensitivity (d’) modulation by alpha activity in the visual network is consistent with our previous report, that stronger pre-stimulus alpha activity in visual areas most responsive to the gratings were associated with lower sensitivity in the detection task.* *Here, we extend this finding by showing that this modulation was only present at times when the visual state was on, that is, when it dominated. It is worth noting that the previous report used localizer trials to identify ROIs most responsive in encoding the stimulus of interest, whereas the current work started by first identifying the alpha networks most active during the task. Convergence of results based on these two different approaches further corroborates our findings on sensitivity. The observed criterion (c) modulation by pre-stimulus alpha activity in the sensorimotor network was unanticipated.*

In addition, as currently written, there are a few places in the text where the conclusions are stated in a way that, while not inaccurate, gives the impression that this is either novel data, or an analysis that is independent from prior analyses of the same data. For example, statements like "our results not only replicate [...] our previous findings" (discussion p2), and "the observed criterion modulation [...] is consistent with our previous observation," risk giving the impression that this is an independent replication. It may be worth reviewing the document with an eye toward revising these types of statements to limit that risk. For example, a simple modification like "our results [using this approach] not only replicate our previous findings" would help clarify the relation between this data and prior work, particularly for a more casual reader.

Great suggestion! We agree it is important to remind our readers that the current study is a re-analysis of our previous work. To this aim, we made the following changes.

In the first paragraph of the Results section:

*Using the exact same data, we have previously shown that our priming manipulation worked, i.e., participants were significantly more liberal in reporting “grating present” in the liberal condition than in the conservative condition (p < 10-6), with no significant difference in sensitivity (12).*

At the start of the second paragraph of the Discussion section:

*By re-analyzing the data using a network-based approach, the current study not only replicates but also extends our previous findings using an ROI-based approach to link alpha activity to perception (12).*

[Results/Interpretation]

[Figure 4 and associated results] If I understand the approach correctly, each of the time-points represented in Figure 4 may be associated with a different number of trials depending on how often a particular state is "on/off." Would it be possible to add some representation of how much data contributes to the model at each timepoint (perhaps a supplemental figure with time-series, like figure 4, but in which the y-axis is just a count)?

Indeed, each time-point in Figure 4 may be associated with a different number of trials depending on how often a particular state is on (or off). We have added the figure as the reviewer suggested as supplemental figure 3 to make this obvious and transparent.

***Figure S3.*** *The number of trials used per GLMM, for each time point and each state. Upper row:* $A=0$*; lower row:* $A=1$*.*

[Figure 4 and associated results] For the time series presented in figure 4, could the authors clarify how time relates to the window over which power is extracted for each point (e.g. are values centered, such that -250ms represents power over the window from -500 to 0ms)? It would be helpful to understand the extent to which apparent prestimulus effects (e.g. for state 7) can be attributed to spread of post-stimulus effects to the prestimulus interval.

The reviewer’s interpretation of the time window is correct and we have now clarified this in the Methods section (see below). The estimated beta_d’ (or beta_c) shown at time *t* corresponds to the sensitivity effect (or criterion effect) of alpha power estimated within the [*t*-0.25, *t*+0.25] s time window. Therefore, the pre-stimulus sensitivity modulation observed for state 7 (starting around 0.3 s before stimulus onset) is unlikely a spread of post-stimulus effects to the pre-stimulus interval.

*With these alpha peak frequencies at hand, the alpha power time courses were computed by first applying the first taper of the Slepian sequence and then a fast Fourier transform to short sliding windows of the critical nodes’ time series data (500 ms in length centered at the time point of interest, sliding in 40 ms steps, ±3 Hz spectral smoothing).*

[Minor: Figure 4 and associated results/interpretation] Throughout the results/discussion, I don't think there is a direct statement regarding the directionality of the effects represented by Figure 4, state 7 (statements are more along the lines of, for example, "Our results showed that ongoing alpha power fluctuation in the visual state (i.e., state 7) significantly modulated sensitivity"). Since the modeling approach may take readers time to parse, it would be helpful to clearly state directionality in both the results and discussion (i.e., that reduction in alpha power when the 'visual' state is on is associated with increased sensitivity).

We thank the reviewer for pointing this out. Indeed, directionality of the effects is critical in the current work, and deserves more highlight and discussion. We have updated the manuscript as suggested:

In the Results section:

*Our results showed that ongoing alpha power fluctuation in the visual state (i.e., state 7) significantly modulated sensitivity only when the state was on (p < 10^-4^), but not when the state was off (interaction effect p < 10^-4^). The estimated* $\beta_{d^{'}, A=1}$ *was negative, suggesting that stronger alpha activity in the visual network was associated with lower sensitivity.*

Also, in the Results section:

*This effect corresponded to a cluster around -250 ms (locked to stimulus onset), suggesting it is most likely pre-stimulus. That is, when the sensorimotor network dominates, stronger pre-stimulus alpha power in this network resulted in more conservative decisions.*

In the Discussion section:

*The observed sensitivity (d’) modulation by alpha activity in the visual network is consistent with our previous report, that stronger pre-stimulus alpha activity in visual areas most responsive to the gratings was associated with lower sensitivity in the detection task. Here, we extend this finding by showing that this modulation was only present at times when the visual state was on, that is, when it dominated.*

Very Minor (Method) - When discussing power spectral decomposition (p3 under Hidden Markov Modeling), the term 'cross PSD' is introduced without definition. Could the authors provide a sentence of elaboration in this paragraph to help the naive reader more directly follow the method?

We have clarified the term ‘cross PSD’ in the updated version:

*Seven Slepian tapers were fitted to the parcel time series data, conditioned on the on-off status of each state, resulting in participant-, parcel-, and state-specific PSDs and cross PSDs (i.e., the frequency-dependent covariance between pairs of the parcel-specific PSDs within each state and each participant).*

Very Minor (Results) - for the rmANOVA of alpha center frequency by state, the authors describe excluding subjects from the main analyses if an alpha component is absent in one or more states for that subject (resulting in 22 subjects). The pairwise follow-up tests suggest subjects were added back into the analyses based on whether alpha-related data existed for a subject in both states. If I'm following the approach correctly, it might help the reader to add a little clarification on the total number of participants contributing to each pairwise comparison. It would also be reasonable here to add a little note with specifics on the type of post-hoc test performed (or multiple comparison correction, if these are simple t-tests performed outside of the rmANOVA context).

The reviewer is correct in pointing out the slight variations in subject number in the post-hoc paired-sample t-tests. We have updated the text to make this clear:

*Paired-sample t-tests on peak frequency showed that state 7 exhibited a significantly higher peak frequency (group-averaged center frequency CF_7_ = 11.02 Hz, alpha oscillatory component identified in N_7_ = 28 participants) compared to state 2 (CF_2_ = 9.83 Hz, N_2_ = 28; state 7 vs. state 2: N = 27, t(26) = 3.659, p = 0.001), state 4 (CF_4_ = 10.31 Hz, N_4_ = 28; state 7 vs. state 4: N = 28, t(27) = 3.447, p = 0.002), and state 8 (CF_8_ = 10.17 Hz, N_8_ = 25; state 7 vs. state 8: N = 23, t(22) = 2.399, p = 0.025).*

Reviewer #3: This short report probes the role of alpha networks in perceptual variability using task based MEG and HMM. The authors propose that existing work has focused on whole-brain alpha or alpha in specific regions and thus have missed the existence of multiple alpha networks and their contribution to perceptual decision making. Overall, the paper pursues an interesting idea, but many of the conclusions are not supported by the data and many of methodological decisions are not clearly motivated or data driven as is suggested in multiple sections. Major concerns:

We thank the reviewer for their effort in providing helpful comments.

1.      The arguments made about the functional role of alpha ignore a sizable, significant  aspect of the literature. Virtually no mention is made of widely known alternative views and this really undermines the paper's capacity to have a major impact.

We thank the reviewer for their comment and have taken the opportunity to more broadly discuss the alpha literature in our Introduction and Discussion sections.

In the Introduction:

*While the functional inhibition account has recently been challenged by alternative interpretations, such as those emphasizing alpha’s role in predictive coding (17–19) and signal enhancement (20,21), there is broad consensus that fluctuations in ongoing alpha activity are tightly linked to perceptual variability (1,2).*

In the Discussion:

*Beyond the gain control and gating accounts, there are alternative theories on the functional relevance of alpha oscillations in cognition. For instance, it has been suggested that alpha oscillations prepare the brain for predictable inputs (17,64) and “carry” top-down predictions (18). In our current and previous work (12), however, we found little evidence in support of this view despite prominent criterion modulation by conservative vs. liberal priming. This discrepancy may reflect differences in the nature of predictions across studies, in that predictions about target presence (as in our case) engage different mechanisms than predictions about target content (as in other work) (65). On the other hand, it has been suggested that alpha desynchronization underlies sensory signal enhancement especially during attentional selection (20,21,66). Consistent with this account, we found that alpha desynchronization in the visual network tracked changes in perceptual sensitivity, suggesting alpha’s role in amplifying sensory signal of interest. However, as our design did not allow us to contrast attended and unattended (or distracting) targets, our current results do not directly address alpha’s role in selecting relevant over irrelevant information during cognitive processing.*

*The diversity of existing theories on alpha oscillations likely stems, at least in part, from treating alpha as a unitary phenomenon (35)—an oversimplification often imposed by the limited spatial resolution of EEG and MEG. Our findings, that distinct alpha networks modulate perception via different mechanisms, highlight the need to move beyond global characterizations of alpha activity and instead analyze it at the level of networks of distinct spatiotemporal and functional configurations.*

2.      The alpha range considered is very broad (7-14 Hz) and includes what many would consider aspects of both theta and beta. No justification provided.

We thank the reviewer for pointing this out. When applying the FOOOF algorithm to find alpha oscillatory peaks within the state PSDs, we have indeed used a slightly wider range of 7 to 14 Hz. This range was defined so as to be consistent with previous work (Haegens, Cousijn, Wallis, Harrison, & Nobre, 2014; Zhou, Iemi, Schoffelen, de Lange, & Haegens, 2021). We have now clarified this in the manuscript:

*The range of 7 to 14 Hz was used so as to be consistent with previous work (12,56).*

3.      Voxel sizes are very large for task-based MEG (8 mm), but ultimately they are collapsed into a 52-parcel atlas, which is also very nonspecific and of unclear motivation. Why take such a coarse approach? MEG is known to be more spatially specific than this and averaging over size large areas can lead to inaccurate conclusions.

The reviewer is right that MEG sometimes is reported to be more spatially specific than this. The primary reason of collapsing the source data into a 52-parcel atlas is based on the observation that the overall rank of the MEG data would normally reduce to 60-80 dimensions after pre-processing (Ferrante et al., 2022). That said, one could still consider using an atlas with more parcels (i.e., spatially more specific), but this makes it difficult to guarantee that the inferred source activity of each parcel is (maximally) orthogonal to each other. Also, when applying the TDE-HMM with the current setting (i.e., with 15 embedding), including $n$ more parcels will lead to an addition of $n*15$ channels to the input matrix, which will cost significantly more time in fitting the HMM. Given these two reasons, we and other researchers (Guichet, Harquel, Achard, Mermillod, & Baciu, 2025; Kohl et al., 2024) believe the current 52-parcel atlas represents the “sweet spot” balancing spatial specificity and computational cost.

4.      Many elements of the signal processing narrow the possible contributions of other spectral windows and neural responses, with the net impact that the results feel forced in many respects.  I don't see how this could be defined as a data driven approach.

Most importantly, our conclusions stand regardless of whether we name our approach “data-driven” or “hypothesis-driven”. In fact, we only used the term “data-driven” for the HMM approach, which helped us to find frequency-specific large-scale brain networks. As opposed to conventional methods using some established (most likely with MRI) atlas to define networks, the TDE-HMM uses the source reconstructed time series data to configure frequency-specific networks in the current data. Given how the HMM was fitted and how the best model was selected, we believe the use of “data-driven” is an appropriate description of the approach, specifically to reveal networks in the data.

Signal processing steps to then zoom in on alpha networks are justified given our hypothesis-based focus on the alpha band in this study, but again, note these steps *follow* the HMM identification of networks in the full time series data. That is, if no alpha networks had been present, our approach would not artificially have revealed these. Note that for each state, spectra are computed for the [1, 45] Hz frequency range; possible contributions of other spectral ranges would have been revealed here if present in the data.

5.      The "states" generally appear to be well known brain responses that occur at different times in perceptual decision tasks, including the anterior shifting wave following initial sensory activation at the midline and spreading to lateral occipital and parietal, as well as the motor response.  The motor response would typically be more beta dominant but the authors forced beta's contribution out of the model so the residual appears like a "alpha network." Again, this is problematic as it significantly hampers the interpretation.

The reviewer’s observation is valid that the occurrence of the states reflects well known brain responses in perceptual decision tasks. Yet, we believe this is reassuring, rather than concerning, from the analysis perspective. One should be extremely worried if this was not the case, because results on brain function revealed with different methods are expected to and should converge.

We thank the reviewer for pointing out beta’s contribution to the motor response. In fact, the FOOOF algorithm found two oscillatory components in the motor state’s PSD (see figure below, the black line denotes the original PSD, red line the reconstructed PSD by FOOOF, dashed blue line the 1/f fit by FOOOF, and the green shaded area and lines denote the oscillatory components detected by FOOOF), with one centering at 9.6 Hz and the other centering at 18.8 Hz, suggesting the coexistence of alpha and beta components.

The reviewer’s claim that we “forced beta’s contribution out of the model” appears to be based on a misconception that the modelling was performed on filtered or otherwise pre-processed data focused on the alpha range — we want to emphasize that this is not the case. The HMM fitting was done on broadband time series data (low-passed at 125 Hz), after which we computed power and coherence spectra to specifically identify alpha networks. Thus, the model could (and did) return networks in other spectral bands, but given the research question at hand here we focused on alpha networks in our further analysis.

6.      The figures are low quality. The glass brains with dots marking parcels and lines connecting them are severely dated and don't provide significant insight on the data.

We thank the reviewer for their feedback on the aesthetics. The glass brains with dots and lines are not our invention, but a common norm used to show connectivity profiles in similar/relevant work. We believe using these glass brains allows better comparison across studies. However, we are open to suggestions of additional helpful ways to visualize these data.

As for the figures’ resolution, this may have been a result of compression of the figures in the pdf generated for review by the editorial manager system (note that the original high-resolution figures can be downloaded by clicking the link in the top-right corner of the respective pages in that pdf), and we will ensure to upload high-quality figures to the journal’s editorial system.

**REFERENCES**

Ferrante, O., Liu, L., Minarik, T., Gorska, U., Ghafari, T., Luo, H., & Jensen, O. (2022). FLUX: A pipeline for MEG analysis. *NeuroImage*, *253*(119047), 119047. doi: 10.1016/j.neuroimage.2022.119047

Gohil, C., Huang, R., Roberts, E., van Es, M. W. J., Quinn, A. J., Vidaurre, D., & Woolrich, M. W. (2024). Osl-dynamics, a toolbox for modeling fast dynamic brain activity. *ELife*, *12*. doi: 10.7554/eLife.91949

Guichet, C., Harquel, S., Achard, S., Mermillod, M., & Baciu, M. (2025). Lifespan oscillatory dynamics in lexical production: A population-based MEG resting-state analysis. *Imaging Neuroscience*, *3*. doi: 10.1162/imag_a_00551

Haegens, S., Cousijn, H., Wallis, G., Harrison, P. J., & Nobre, A. C. (2014). Inter- and intra-individual variability in alpha peak frequency. *NeuroImage*, *92*, 46–55. doi: 10.1016/j.neuroimage.2014.01.049

Kohl, O., Gohil, C., Zokaeia, N., Hu, M. T., Nobrea, A. C., Woolrich, M., & Quinn, A. (2024). Changes in sensorimotor network dynamics in resting-state recordings in Parkinson’s Disease (p. 2024.01.22.24301617). doi: 10.1101/2024.01.22.24301617

Quinn, A. J., Vidaurre, D., Abeysuriya, R., Becker, R., Nobre, A. C., & Woolrich, M. W. (2018). Task-Evoked Dynamic Network Analysis Through Hidden Markov Modeling. *Frontiers in Neuroscience*, *12*, 603. doi: 10.3389/fnins.2018.00603

van Es, M. W. J., Higgins, C., Gohil, C., Quinn, A. J., Vidaurre, D., & Woolrich, M. W. (2023). Large-scale cortical networks are organised in structured cycles (p. 2023.07.25.550338). doi: 10.1101/2023.07.25.550338

Vidaurre, D., Hunt, L. T., Quinn, A. J., Hunt, B. A. E., Brookes, M. J., Nobre, A. C., & Woolrich, M. W. (2018). Spontaneous cortical activity transiently organises into frequency specific phase-coupling networks. *Nature Communications*, *9*(1), 2987. doi: 10.1038/s41467-018-05316-z

Zhou, Y. J., Iemi, L., Schoffelen, J.-M., de Lange, F. P., & Haegens, S. (2021). Alpha oscillations shape sensory representation and perceptual sensitivity. *The Journal of Neuroscience: The Official Journal of the Society for Neuroscience*. doi: 10.1523/JNEUROSCI.1114-21.2021
