# Supplementary material for: Distinct alpha networks modulate different aspects of perceptual decision-making
Source: PLoS Biol. 2025 Oct 22;23(10):e3003461. doi: 10.1371/journal.pbio.3003461 (PMC12561919; doi:10.1371/journal.pbio.3003461)
Supplement: Attachment — Submitted filename: PLOS_Bio_FinalRevision.docx [file pbio.3003461.s010.docx]

Dear editors and reviewers,

Thank you for your time and effort handling our article. Please see below for our point-to-point reply (in blue) to each comment/suggestion.

**Financial disclosure statement:

-- Please add links to the funding agencies in the Financial Disclosure statement in the manuscript details.

We have added the links to our funders via the “Financial Disclosure” section in the Editorial Manager (EM).


**Ethics: 

 -- We recognize that your study analyzes previously collected data. However, because this data is from human subjects, we request that you include an Ethics statement explaining the ethical guidelines that governed the original data collection (or indicate that your study was explicitly exempted if applicable).

-- Please include information about the form of consent (written/oral) given for research involving human participants. All research involving human participants must have been approved by the authors' Institutional Review Board (IRB) or an equivalent committee, and must have been conducted according to the principles expressed in the Declaration of Helsinki.

-- The Ethics statement needs to be a separate, independent (and the first) subheading in the Material & Methods section. It must  include the full name of the IACUC/ethics committee that reviewed and approved the animal care and use, as well as the protocol/permit/project license number.  <https://journals.plos.org/plosbiology/s/ethical-publishing-practice>

Thanks for the suggestions. We updated our answer in the EM and added the following Ethics statement in our manuscript.

**Ethics Statement**

Ethical approval for the study was granted by the local ethics committee (Commissie Mensgebonden Onderzoek, Arnhem-Nijmegen). All participants provided written informed consent.


**Data:

-- We see that you wrote that data access will be provided after acceptance. Please upload these data and make them available.

-- In particular, please supply the numerical values either in a supplementary excel file or as a permanent DOI’d deposition for Figure 3B.

NOTE: the numerical data provided should include all replicates AND the way in which the plotted mean and errors were derived (it should not present only the mean/average values).

-- Please cite the location of the data clearly in all relevant main and supplementary Figure legends, e.g. “The data underlying this Figure can be found in S1 Data” or “The data underlying this Figure can be found in <https://doi.org/10.5281/zenodo.XXXXX>”

-- Supplementary files (e.g., excel). Please ensure that all data files are uploaded as 'Supporting Information' and are invariably referred to (in the manuscript, figure legends, and the Description field when uploading your files) using the following format verbatim: S1 Data, S2 Data, etc. Multiple panels of a single or even several figures can be included as multiple sheets in one excel file that is saved using exactly the following convention: S1_Data.xlsx (using an underscore).

We can confirm that all data are already made available. Readers can follow the data access guidelines on the Donders Repository to download the raw MEG data, and follow the Zenodo guidelines to download the TDE-HMM results and code to reproduce these results.

We have uploaded the above-mentioned values as a supplementary Excel file to the EM system.

We have updated the Figure legends as suggested and uploaded the corresponding data files to the EM system.


 **Code availability:

-- Per journal policy, if you have generated any custom code during the course of this investigation, please make it available without restrictions. Please ensure that the code is sufficiently well documented and reusable, and that your Data Statement in the Editorial Manager submission system accurately describes where your code can be found.
-- Please note that we cannot accept sole deposition of code in GitHub, as this could be changed after publication. However, you can archive this version of your publicly available GitHub code to Zenodo. Once you do this, it will generate a DOI number, which you will need to provide in the Data Accessibility Statement (you are welcome to also provide the GitHub access information). See the process for doing this here: <https://docs.github.com/en/repositories/archiving-a-github-repository/referencing-and-citing-content>

We have updated the Data Statement in the EM submission system.

Data (both behavioral and MEG) are available from the Donders Repository at http://doi.org/10.34973/w1k5-sm41. HMM results and the code to reproduce the results can be found via https://doi.org/10.5281/zenodo.17217503.


**Abstract:

-- Please note that per journal policy, the model system/species studied should be clearly stated in the abstract of your manuscript.

We have updated the abstract and stated the model species in the Abstract.

Here, to characterize how different alpha networks influence perceptual decision-making, we analyzed magnetoencephalography (MEG) data recorded while human participants performed a visual detection task with threshold-level stimuli.
